# Breast Feeding Practices and Newborn Care in Rural Areas: A Descriptive Cross-Sectional Study

**DOI:** 10.4103/0970-0218.55292

**Published:** 2009-07

**Authors:** K Madhu, Sriram Chowdary, Ramesh Masthi

**Affiliations:** Department of Pharmacology, St Johns Medical College, Bangalore - 34, India; 1 Kempegowda Institute of Medical Sciences, Bangalore - 56, India; 2 Department of Community Medicine, Kempegowda Institute of Medical Sciences, Bangalore, India

**Keywords:** Breastfeeding, duration, initiation, rural areas

## Abstract

**Context:**

Breastfeeding practices play an important role in reducing child mortality and morbidity. This study was aimed to describe the breastfeeding practices prevalent in rural areas.

**Objectives:**

The primary objective of this study was to describe the breastfeeding and newborn care practices in rural areas and the secondary objective was to describe the factors affecting the initiation and duration of breastfeeding.

**Settings and Design:**

The study was conducted in primary health care center (PHC) that is attached to a medical college in Kengeri, rural Bangalore, Karnataka.

**Materials and Methods:**

Mothers with children who were 9 months old who came to the PHC for measles vaccination were included in the study and data was collected using the pre-tested questionnaire on breastfeeding and newborn practices.

**Results:**

Our study shows 97% of the mothers initiated breastfeeding, 19% used pre lacteal feeds, 90% had hospital deliveries and 10% had home deliveries, and 50% used a house knife to cut the umbilical cord among home deliveries.

**Conclusions:**

This study emphasizes the need for breastfeeding intervention programs especially for the mother during antenatal and postnatal check-ups and practices like discarding the colostrum and early/late weaning are still widely prevalent and need to be addressed.

## Introduction

Breastfeeding is one of the most important determinants of child survival, birth spacing, and prevention of childhood infections. The importance of breastfeeding has been emphasized in various studies.(1,2) The importance of exclusive breastfeeding and the immunological and nutritional values of breast milk has been demonstrated.(3,4)

The beneficial effects of breastfeeding depend on breastfeeding initiation, its duration, and the age at which the breast-fed child is weaned.(5) Breastfeeding practices vary among different regions and communities. In India, breastfeeding in rural areas appears to be shaped by the beliefs of a community,(1) which are further influenced by social, cultural, and economic factors. Hence, the study with these relationships helps in orienting the breastfeeding promotional activities and for preventing a decline in initiation and duration of breastfeeding practices.

In this study, we are trying to look at the demographic variables, breastfeeding, and newborn care practices. Information about newborn care and breastfeeding practices in the rural population will be useful for policy makers and for interventional programs.

## Materials and Methods

This cross-sectional study was conducted at a primary health care center (PHC) that is attached to a rural health care training center in Kengeri, rural Bangalore, Karnataka from January 2006 to April 2006 for the period of 4 months. The Kengeri PHC catchment area has a population of 30,000. Mothers with infants aged 9 months who came to the PHC for measles vaccination were included in the study. Verbal consent was obtained. Those who were not willing to participate were excluded. All the mothers agreed to participate in the study.

A pre-tested questionnaire was used.(2) Over a period of 4 months, all consecutive mothers coming to the rural health care center were interviewed until the sample size of 100 was reached.

The pre-tested questionnaire included various factors that had a potential effect on the initiation and duration of breastfeeding practices [Table 1]. The questionnaire included socio-economic and demographic data, details on the initiation and duration of breastfeeding, details on artificial feeding and weaning practices, and newborn care practices. A pre-test run was done to validate the questionnaire. For socio-economic status, a standard of living index(6) was used that can be used for both urban and rural backgrounds. There were a total of 33 questions on breastfeeding practices and 8 on newborn care practices.

**Table 1:** Factors considered as potential influence on the initiation and duration of breast feeding

Socio demographic profile
Mothers age in years at the time of child birth
Mothers formal education
Mothers number of deliveries
Mothers employment status
Variables relating to medical care during pregnancy and delivery
Number of prenatal checkups
Health personal conducting the prenatal checkups
Health personal responsible for care during child birth
Place of delivery (home, govt. hospital, private facility)
Variables related to pregnancy and the child
Illness during pregnancy reported by the mother
Type of delivery
Child sex
Child's birth weight

Statistical analysis used: Data analysis was done according to descriptive statistics. Results are given in percentages.

## Results

### Socio demographic profile

In our study, the majority of the mothers were between the ages of 21 and 25 years old (60%) and 15 and 20 years old (30%). About 52% of the mothers were illiterate and belonged to a low to medium socio-economic class (55%). A majority of the mothers were primigavidae (65%) and the age at marriage was between 15 and 20 years old (69%). Approximately 11% of the mothers were less than 15 years old at the time of marriage. The majority of the mothers were housewives (69%) and mothers who were employed were 22% [Table 2].

**Table 2:** Percentage distribution of the study population by socio demographic characteristics

Socio demographic profile	Number of mothers in percentage
Mothers age	
<19	33
21-25	49
26-30	14
>30	4
Age of marriage	
<19	69
20-25	29
26-30	1
>30	1
Formal education	
None	52
Primary	42
Secondary/University	6
Mothers employment	
Working	21
Not working	79
Socio economic status	
Low	50
Medium	49
High	1
Parity	
1	48
2	43
3	7
4	2

### Initiation of breastfeeding

Most of the mothers initiated breastfeeding (97%) and the other 3% were not able to initiate due to separation from mother (2%) or due to advice from the mother-in-law (1%).

A total of 44% of the mothers initiated breastfeeding within 30 minutes with home delivery and 38% with Caesarean section. There was a delay of 2 to 3 hrs in feeding. A total of 19% of the mothers in our study didn't breastfeed even after 24 hours after the delivery. They were given pre lacteal feeds and discarded the colostrum. A total of 13% of the babies were fed with sugar water alone for more than 48 hours. Honey (6%) and ghee (3%) were also commonly used pre lacteal feeds.

### Duration of breastfeeding

**Figure 1:**
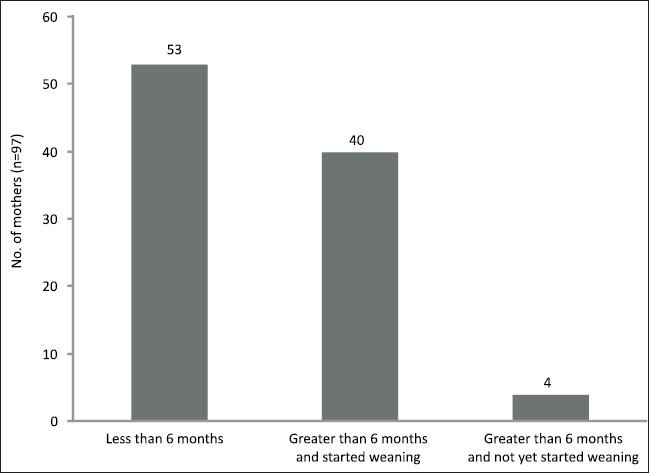
Duration of breast feeding

Only 40% of the mothers did the exclusive breastfeeding until 6 months and started weaning after 6 months [Figure 1]. A total of 53% of the mothers in our study prematurely started weaning the child. A majority of the mothers started weaning at the age of 3 to 4 months. The most common reason given for the start of supplementary feeding was insufficient milk (92%; 49 out of 53).

Only 4 mothers continued to breastfeed the baby even at 9 months. A total of 84% of the mothers followed on-demand feeding practices and rooming in. Cow's milk (26%) was the most common food used for the infants who were breastfed less than 6 months followed by ‘ragi sari’. Ragi sari is crushed millet and is given mixed with milk and water. Only 10 mothers (19%) used commercial formula. Cow's milk was diluted by the mothers before administration.

**Figure 2:**
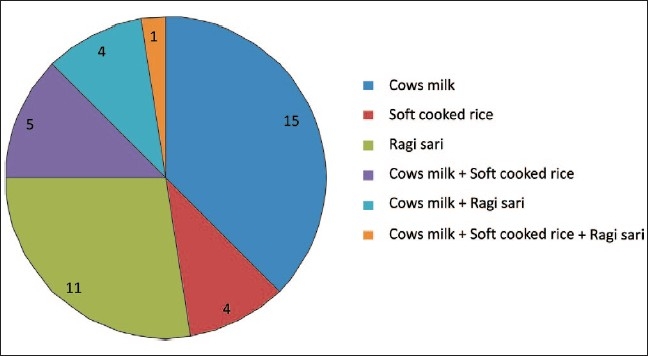
Types of weaning foods used

Among the mothers who started weaning exclusive breastfeeding after 6 months (53%), cow's milk was the most common weaning food (28.3%) and the next common food used was ragi sari (20%) [Figure 2].

A total of 97% of the mothers went for at least two antenatal check-ups, whereas, only approximately 30% went for post natal check-ups.

The information about weaning foods and the time to start weaning was obtained from the doctors by 42% of the mothers, 21% of the mothers obtained this information from their mothers-in-law, and the remaining received the information from neighbours.

### Newborn care practices

A total of 90% of the deliveries were hospital deliveries and 10% were home deliveries. The care provided during the home deliveries was mainly given by an untrained birth attendant (40%). A household knife (50%) was used to cut the umbilical cord in five home deliveries. In both in-hospital and home deliveries, nothing was applied for umbilical cord dressing (67%). Talcum powder (10%) and turmeric was used by some mothers for cord dressing. A total of 16% of the mothers still practiced branding of the child for illness. A total of 93% of the children received all vaccinations needed according to the national immunization schedule.

## Discussion

### Initiation of breastfeeding

Women in rural areas have a very positive attitude towards initiation of breastfeeding.(7) In this study, almost all the women had initiated breastfeeding and continued to breastfeed beyond 9 months. Other studies conducted in rural areas show that almost all the mothers initiate breastfeeding.(8) Urban areas in the other studies also show a similar pattern.(9)

Breast milk should be initiated within 30 minutes of delivery.(10) The delay in initiation will lead to a delay in the development of oxytocin reflexes, which are very important for the contraction of the uterus and the breast milk reflex. In our study, almost all the mothers initiated breastfeeding within 1 hour of childbirth, which is a good practice. Studies comparing the early onset of breastfeeding on the development of newborns and on their mothers and those studies in which breastfeeding had begun on the 6^th^ hour after birth show that the earlier breastfeeding begins, the earlier and more effective the consolidation of the process and therefore a better impact on the after-birth period, which helps in the earlier initiation of the secretion of breast milk.(11)

Pre lacteal feeds should not be given but still the majority of mothers give either sugar water or honey. Discarding the colostrum is still practiced widely. The colostrum is rich in vitamins, minerals, and immunoglobulins that protect the child from infections.(12) Discarding the colostrum and feeding the child with sugar water, honey, or ghee makes the child vulnerable to infections. Other studies have also found similar practices in the community and it is largely influenced by the relatives and the primary care providers during childbirth.(13)

### Duration of breastfeeding

Exclusive breastfeeding should be continued for 6 months.(14) It protects the child from malnutrition, infections, and helps the overall development of the child.(3,4) Only 40% of the mothers were doing exclusive breastfeeding, the remaining 60% of the mothers were not. They prematurely start weaning the child, which may lead to the development of infections and may have a long-term effect on the physical growth of the child.(15) The main reason given for the mother to start weaning early was insufficient milk, which may be due to the early age of marriage (those who were younger than 19 years old) and early childbirth. Studies indicated that adolescents breastfeed less often than adults and they hold positive and negative attitudes toward breastfeeding that influence decision-making and breastfeeding.(16)

In this study, a majority of the mothers had at least two antenatal check-ups and most of the antenatal check-ups were done by a doctor. The mothers who were attending antenatal check-ups during the duration of breastfeeding did not vary. This may be due to the lack of breastfeeding information given to the mothers during antenatal check-ups.

Postnatal check-ups were not attended by a majority of the mothers. It may also have contributed toward the early weaning or late weaning practices. The importance of the intervention in the form of teaching breastfeeding techniques had a positive outcome in the previous studies.(17)

Most of the mothers received information regarding breastfeeding practices from their doctors. The mothers who went to government doctors exclusively breastfed their babies. In contrast, the mothers who went to the private doctors started weaning early. The development of counselling skills among doctors helps in conveying the right message to mothers about breastfeeding and weaning practices.(18)

The influence of the mother-in-law and self assumption about lack of milk for the baby are sited as major reasons for early weaning and late weaning. Other studies have also found similar influences of the mother-in-law and neighbours regarding exclusive breastfeeding.(13)

Regarding the neonatal care practices, hospital deliveries outnumber the home deliveries. This may be due to improved access to health care facilities in the region. The majority of the home deliveries were attended by untrained dai and they used household knives for cord cutting and did not observe aseptic precautions like a clean home delivery kit during delivery. The application of talcum powder on the cord stump often led to infection(19) and was responsible for the ill health of the newborn, while tetanus neonatorum could be directly attributed to the practice of applying cow dung on the cord stump but the practice is found to be very less.

The mothers who did not come to the primary health care center for vaccinations were not included in this study. The mothers who went to privative clinics or who did not come for vaccinations might be different. Sample size is also a limiting factor.

## Conclusions

This study emphasizes the need for breastfeeding intervention programs especially for the mother during antenatal and postnatal check-ups. The information regarding the advantages and duration of breastfeeding needs to be provided for the community as a whole. Practices such as discarding the colostrum and early/late weaning should be discouraged. Training for the traditional birth attendants and maintaining aseptic precautions with the use of clean delivery kits and community-based health education programs is needed.

## References

[1] Iskandar MB, Costello C, Nasution Y (1990). Initiation and Duration of Breast feeding in Indonesia. Asia Pac Popul J.

[2] Bautista LE (1997). Factors associated with initiation of breast feeding in the domician Republic. Rev Panam Salud Publica.

[3] Arifeen S, Black RE, Antelman G, Baqui A, Caulfield L, Becker S (2001). Exclusive breast-feeding reduces acute respiratory infection and diarrhea deaths among infants in Dhaka slums. Pediatrics.

[4] Dewey KG, Cohen RJ, Brown KH, Rivera LL (2001). Effects of exclusive breast-feeding for four versus six months on maternal nutritional status and infant motor development: Results of two randomized trials in Honduras. J Nutr.

[5] Victora CG, Smith PG, Vaughan JP, Nobre LC, Lombardi C, Teixeira AM (1987). Evidence for protection against infant deaths from infectious diseases in Brazil Lancet.

[6] (2004). Standard of living index, NFHS -3 report.

[7] Chandrashekar S, Chakladar BK, Rao RS (1995). Infant feeding--knowledge and attitudes in a rural area of Karnataka. Indian J Pediatr.

[8] Benakappa DG, Raju M, Shivananda, Benakappa AD (1989). Breast-feeding practices in rural Karnataka (India) with special reference to lactation failure. Acta Paediatr Jpn.

[9] Chandrashekhar TS, Joshi HS, Binu V, Shankar PR, Rana MS, Ramachandran U (2007). Breast-feeding initiation and determinants of exclusive breast-feeding: A questionnaire survey in an urban population of western Nepal. Public Health Nutr.

[10] World Health Organisation (1989). UNICEF, Ten steps to promote successful breastfeeding Mother and Child Health Division.

[11] Iarukov A, Nin'o A, Iarukova N, Doicheva E, Kolev D (1992). The early breast feeding of newborn infants. Akush Ginekol (Sofiia).

[12] Davies MC, Arinolan G, Sanusin R, Osotimehin B (2006). Immunoglobulin classes and nutritional factors in plasma and breast milk of lactating mothers in Nigeria. Iran J Immunol.

[13] Sharma M, Kanani S (2006). Grandmothers' influence on child care. Indian J Pediatr.

[14] Kramer MS, Kakuma R (2001). The optimal duration of exclusive breastfeeding: A systematic review.

[15] Hop LT, Gross R, Giay T, Sastroamidjojo S, Schultink W, Lang NT (2000). Premature complementary feeding is associated with poorer growth of vietnamese children. J Nutr.

[16] Wambach KA, Cole C (2000). Breastfeeding and adolescents. J Obstet Gynecol Neonatal Nurs.

[17] Prasad B, Costello AM (1995). Impact and sustainability of a “baby friendly” health education intervention at a district hospital in Bihar, India. BMJ.

[18] Neifert MR (1999). Clinical aspects of lactation: Promoting breastfeeding success. Clin Perinatol.

[19] Osrin D, Tumbahangphe KM, Shrestha D, Mesko N, Shrestha BP, Manandhar MK (2002). Cross sectional, community based study of care of newborn infants in Nepal. BMJ.

